# The Role of Liver in Determining Serum Colon-Derived Uremic Solutes

**DOI:** 10.1371/journal.pone.0134590

**Published:** 2015-08-10

**Authors:** Cheng-Jui Lin, Tai-Cherng Liou, Chi-Feng Pan, Pei-Chen Wu, Fang-Ju Sun, Hsuan-Liang Liu, Han-Hsiang Chen, Chih-Jen Wu

**Affiliations:** 1 Division of Nephrology and Department of Internal Medicine, Mackay Memorial Hospital, Taipei, Taiwan; 2 Division of Gastroenterology and Department of Internal Medicine, Mackay Memorial Hospital, Taipei, Taiwan; 3 Mackay Junior College of Medicine, Nursing and Management, Taipei, Taiwan; 4 Institute of Biotechnology, National Taipei University of Technology, Taipei, Taiwan; 5 Department of Medicine, Mackay Medical College, New Taipei City, Taiwan; 6 Department of Medical Research, Mackay Memorial Hospital, Taipei, Taiwan; 7 Graduate Institute of Medical Science, Taipei Medical University, Taipei, Taiwan; University of Valencia, SPAIN

## Abstract

Evidence has shown that indoxyl sulfate (IS) and p-cresyl sulfate (PCS) may be alternative predictors of clinical outcomes in chronic kidney disease (CKD). Both toxins are derived from the gastrointestinal tract and metabolised in the liver. However, it is unclear whether the liver affects the production of IS and PCS. Here, we explore the association between IS and PCS levels in liver cirrhosis and a CKD-based cohort (N = 115). Liver and kidney function was assessed and classified by a Child-Pugh score (child A–C) and a modified version of the Modification of Diet in Renal Disease (MDRD) equation (Stages 1–4), respectively. An animal model was also used to confirm the two toxin levels in a case of liver fibrosis. In patients with early liver cirrhosis (child A), IS and PCS were significantly associated with CKD stages. In contrast, serum IS and PCS did not significantly change in advanced liver cirrhosis (child C). A stepwise multiple linear regression analysis also showed that T-PCS was significantly associated with stages of liver cirrhosis after adjusting for other confounding factors (B = -2.29, *p* = 0.012). Moreover, the serum and urine levels of T-PCS and T-IS were significantly lower in rats with liver failure than in those without (*p*<0.01, *p*<0.05 and *p*<0.01, *p*<0.05, respectively). These results indicated that in addition to the kidneys, the liver was an essential and independent organ in determining serum IS and PCS levels. The production rate of IS and PCS was lower in patients with advanced liver cirrhosis.

## Introduction

Metabolic disarray in chronic kidney disease (CKD) starts from the moment of progressive loss of kidney function as a result of the accumulation of a large number of solutes that are normally excreted in urine. [1]. These retained uremic solutes are preferentially classified into three kinds of groups according to their physicochemical characteristics. [2]. Some compounds, such as urea, can be removed through dialysis. Many other retained compounds cannot. [3–5]. Protein-bound uremic toxins, including indoxyl sulphate (IS) and p-cresyl sulphate (PCS), belong to this group because high albumin binding limits their clearance. Previous in vitro studies have shown that the two toxins were capable of inducing the production of reactive oxygen species[6–8], and had a detrimental effect on endothelial function. [9–11]. This may further contribute to negative clinical outcomes in CKD, including infection-related hospitalisation, [12,13] renal function progression, [14,15] cardiovascular events and all-cause mortality. [13,16–18].

However, both IS and PCS emanate from the gastrointestinal tract. The precursors of the two solutes were generated by metabolism of dietary protein by the intestinal flora. Subsequently, they were metabolised by the cytosolic enzymes of hepatocyte, before finally being secreted due to systemic circulation. Serum levels of IS and PCS depended primarily upon the severity of CKD, [14,19] and neither could be removed efficiently by haemodialysis or peritoneal dialysis. [20,21]. However, little evidence exists to demonstrate whether liver function is able to affect IS and PCS concentrations.

Thus, we propose a thesis that there is a close association between liver function and serum IS and PCS levels. Our study’s purpose is to investigate whether or not liver function is able to determine serum concentrations of IS and PCS in a cohort with liver cirrhosis.

## Results

Table 1 shows the demographic and clinical characteristics of patients. In all, 115 stable CKD patients (Stages 1–4) with liver cirrhosis (child A–C) were recruited for the study. The participants consisted of 65.2% males and 34.8% females with a mean age of 61.7 ± 11.6 years. Overall, 35.7% of the patients had HBV, 30.4% of the patients had HCV and 28.7% of the patients had alcoholic liver disease. The average free and total serum IS was 0.04 ± 0.05, 1.60 ± 2.95 mg/L, respectively. The average free and total PCS was 0.10 ± 0.23, 3.51 ± 6.37 mg/l, respectively. Pearson’s analysis was used to determine the correlation between liver cirrhosis and protein-bound uremic toxins at different CKD stages, as shown in Table 2. In patients with early CKD (Stages 1–3), most serum protein-bound uremic toxins did not significantly correlate to a liver cirrhosis stage. However, total IS (T-IS) and total PCS (T-PCS) (r = -0.75, *p* = 0.003, r = -0.72, *p* = 0.005, respectively) were negatively associated with liver stage in advanced CKD (Stage 4).

**Table 1:** Baseline characteristics of the study patients.

Variables	ALL (n = 115)
**Age (yr)**	**61.7±11.6**
**Male (%)**	**65.20%**
**Diabetes Mellitus (%)**	**44.30%**
**Hypertension (%)**	**36.50%**
**HBV (%)**	**35.70%**
**HCV (%)**	**30.40%**
**Alcohol (%)**	**28.70%**
**SBP (mmHg)**	**124.1±14.3**
**DBP (mmHg)**	**71.6±11.3**
**CKD stages (%)**	
**1**	**30.40%**
**2**	**36.50%**
**3**	**20.90%**
**4**	**12.20%**
**Liver cirrhosis stages**	
**Child A**	**59.10%**
**Child B**	**28.70%**
**Child C**	**12.20%**
**Medication (%)**	
**OAD**	**44.30%**
**ACEI / ARB**	**24.30%**
**CCB**	**30.40%**
**Anti-viral therapy**	**18.30%**
**Albumin (g/dL)**	**3.2±0.6**
**Hemoglobin (g/L)**	**10.9±2.1**
**Hematocrit (%)**	**32.1±5.7**
**BUN (mg/dL)**	**21.8±20.7**
**Creatinine (mg/dL)**	**1.4±1.6**
**eGFR (ml/min/per1.73 m2)**	**74.5±35.4**
**F-IS (mg/L)**	**0.04±0.05**
**T-IS (mg/L)**	**1.6±2.95**
**F-PCS (mg/L)**	**0.1±0.23**
**T-PCS (mg/L)**	**3.51±6.37**

**Table 2:** Correlation between liver cirrhosis and T-IS, F-IS, T-PCS and F-PCS in different CKD sages by Pearson’s analysis.

	CKD stages									
	stage 1		stage 2		stage 3		stage 4		Total	
	Liver Cirrhosis		Liver Cirrhosis		Liver Cirrhosis		Liver Cirrhosis		Liver Cirrhosis	
	*r*	*p*	*r*	*p*	*r*	*p*	*r*	*p*	*r*	*p*
**T-IS**	-0.39	0.028	-0.02	0.898	-0.24	0.357	-0.75	0.003	-0.19	0.063
**F-IS**	-0.28	0.121	-0.34	0.046	-0.18	0.488	-0.51	0.078	-0.17	0.089
**T-PCS**	-0.02	0.930	-0.32	0.057	-0.50	0.043	-0.72	0.005	-0.25	0.012
**F-PCS**	-0.07	0.722	-0.29	0.085	-0.33	0.196	-0.40	0.178	-0.14	0.157

Table 3 illustrates the correlation between CKD stages and protein-bound uremic toxins in different liver cirrhosis stages. Serum T-IS, free IS (F-IS), T-PCS and free PCS (F-PCS) showed a strong positive correlation with CKD stages in all patients with liver cirrhosis. This trend was also present in patients with child A liver cirrhosis. Only F-IS and F-PCS (*r* = 0.57, *p* = 0.001, *r* = 0.45, *p* = 0.016, respectively) reached a level of significant change in child B liver cirrhosis. There was no obvious difference between serum uremic toxins and CKD stages in patients with child C liver cirrhosis.

**Table 3:** Correlation between CKD stages and T-IS, F-IS, T-PCS and F-PCS in different liver cirrhosis sages by Pearson’s analysis.

	Liver Cirrhosis							
	child A		child B		child C		Total	
	CKD		CKD		CKD		CKD	
	*r*	*p*	*r*	*p*	*r*	*p*	*r*	*p*
**T-IS**	0.60	p<0.001	0.28	0.147	0.14	0.663	0.39	p<0.001
**F-IS**	0.45	p<0.001	0.57	0.001	0.06	0.845	0.36	p<0.001
**T-PCS**	0.64	p<0.001	0.07	0.724	0.41	0.185	0.36	p<0.001
**F-PCS**	0.45	p<0.001	0.45	0.016	0.17	0.602	0.34	p<0.001

**Fig 1:**
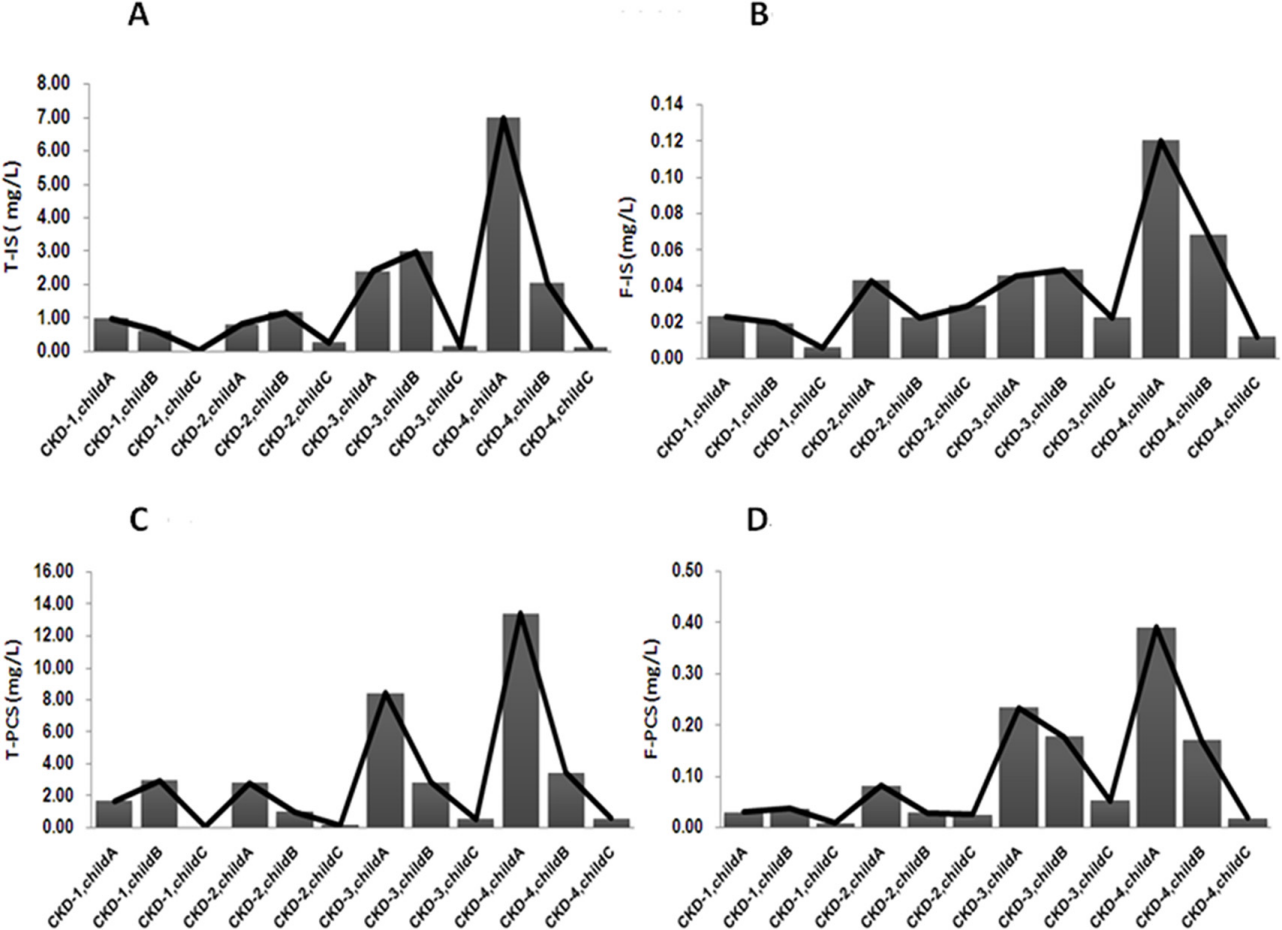
Serum levels of protein-bound uremic toxins (A) T-IS, (B) F-IS, (C) T-PCS and (D) F-PCS in patients with different CKD and liver cirrhosis.

We used a two-way ANOVA with a Least Squares Means test to approach the interaction effect between CKD stages and liver cirrhosis stages. Table 4 outlines the means and interactions in the different subgroups of CKD and liver cirrhosis stages. Only F-PCS did not have an interaction effect between different CKD and liver cirrhosis stage (*p* = 0.115). There was an interaction for F-IS, T-IS and T-PCS (please see Table 5). The results showed that in patients with early CKD or liver cirrhosis, the serum protein-bound uremic toxin concentration did not change significantly. Until renal function declined (advanced CKD Stages 3 and 4), we found that the four toxin concentrations increased, reaching a significant difference as compared to different liver cirrhosis stages. Fig 1 shows the four toxins at different CKD and liver cirrhosis stages.

**Table 4:** The mean of T-IS, F-IS, T-PCS. F-PC in different CKD and liver Cirrhosis stages and their interaction analyzed by two-way ANOVA.

Variables		CKD stages				CKD stage	Liver Cirrhosis	CKD stage ×
						effect	effect	Liver Cirrhosis
		stage 1	stage 2	stage 3	stage 4			effect
	**Liver Cirrhosis**	**mean±SD**	**mean±SD**	**mean±SD**	**mean±SD**			
**T-IS**	**A**	0.97±0.71	0.81±0.67	2.39±1.96	11.97±7.12	p<0.001	p<0.001	p<0.001
	**B**	0.63±0.8	1.16±2.41	2.98±5.1	2.02±0.93			
	**C**	0.02±0.02	0.25±0.22	0.15±0.16	0.12±0.15			
	**Liver Cirrhosis**							
**F-IS**	**A**	0.02±0.01	0.04±0.02	0.05±0.05	0.18±0.21	0.002	0.001	0.014
	**B**	0.02±0.02	0.02±0.02	0.05±0.02	0.07±0.05			
	**C**	0.01±0.01	0.03±0.02	0.02±0.04	0.01±0.01			
	**Liver Cirrhosis**							
**T-PCS**	**A**	1.66±1.96	2.79±3.57	8.41±7.61	22.67±13.74	p<0.001	p<0.001	p<0.001
	**B**	2.93±7.01	0.96±1.46	2.83±2.85	3.41±4.09			
	**C**	0.04±0.04	0.13±0.15	0.51±0.75	0.54±0.82			
	**Liver Cirrhosis**							
**F-PCS**	**A**	0.03±0.02	0.08±0.1	0.23±0.23	0.59±1	0.017	0.007	0.115
	**B**	0.04±0.07	0.03±0.05	0.18±0.2	0.17±0.19			
	**C**	0.01±0.01	0.02±0.04	0.05±0.07	0.02±0.02			

**Table 5:** Interaction analysis between patients with different CKD and liver cirrhosis stages for protein-bound uremic toxins by Least Squares Means.

CKD stage &			1			2			3			4		
**Liver Cirrhosis**			**1A**	**1B**	**1C**	**2A**	**2B**	**2C**	**3A**	**3B**	**3C**	**4A**	**4B**	**4C**
**T-IS**	**3**	**A**				**0.04**								
		**B**												
		**C**												
	**4**	**A**	**p<0.001**	**p<0.001**	**p<0.001**	**p<0.001**	**p<0.001**	**p<0.001**	**p<0.001**	**p<0.001**	**p<0.001**			
		**B**										**p<0.001**		
		**C**										**p<0.001**		
**F-IS**	**3**	**A**												
		**B**												
		**C**												
	**4**	**A**	**p<0.001**	**p<0.001**	**p<0.001**	**p<0.001**	**p<0.001**	**p<0.001**	**p<0.001**	**p<0.001**	**p<0.001**			
		**B**	**0.044**									**p<0.001**		
		**C**										**p<0.001**		
**T-PC**S	**3**	**A**	**p<0.001**	**0.014**	**0.009**	**0.003**	**0.001**	**0.01**						
		**B**												
		**C**							**0.014**					
	**4**	**A**	**p<0.001**	**p<0.001**	**p<0.001**	**p<0.001**	**p<0.001**	**p<0.001**	**p<0.001**	**p<0.001**	**p<0.001**			
		**B**							**0.046**			**p<0.001**		
		**C**							**0.014**			**p<0.001**		
**F-PC**S	**3**	**A**	**0.017**				**0.037**							
		**B**												
		**C**												
	**4**	**A**	**p<0.001**	**p<0.001**	**0.001**	**p<0.001**	**p<0.001**	**0.001**	**0.006**	**0.008**	**0.002**			
		**B**										**0.003**		
		**C**										**0.001**		

In addition, univariate and multivariate (stepwise) linear regression analyses were used to evaluate the relationship between independent variables and uremic toxins (Table 6). In the univariate analysis, T-IS was correlated with age (*p* = 0.01), CKD stage (*p*<0.01), systolic blood pressure (*p* = 0.06), blood urea nitrogen (BUN) (*p*<0.001), creatinine (Cr) (*p*<0.001) and estimated glomerular filtration rate (eGFR) (*p*<0.001); F-IS was associated with CKD stage (*p*<0.001), systolic blood pressure (*p*<0.001), hypertension (HTN) (*p*<0.009), BUN (*p*<0.001), Cr (*p*<0.001) and eGFR (*p*<0.001); T-PCS was correlated with age (*p*<0.001), CKD stage (*p*<0.001), liver cirrhosis stage (*p* = 0.012), systolic blood pressure (*p* = 0.004), HTN (*p* = 0.011), alcohol consumption (*p* = 0.018), BUN (*p*<0.001), Cr (*p*<0.001) and eGFR (*p*<0.001); F-PCS was correlated with CKD stage (*p* = 0.001), systolic blood pressure (*p* = 0.001), HTN (*p* = 0.018), BUN (*p*<0.001), Cr (*p*<0.001) and eGFR (*p*<0.001). After adjusting for other confounding factors using a multivariate analysis, T-IS was only correlated with Cr (*p* = 0.002); F-IS was associated with BUN (*p*<0.001); T-PCS was correlated with age (*p* = 0.018), liver cirrhosis stage (*p* = 0.040), and BUN (*p*<0.001); F-PCS was associated with BUN (*p*<0.001).

**Table 6:** Univariate and multivariate (Stepwise) linear regression analyses for evaluating the relationship between independent variables and clinical outcomes in CKD and liver cirrhosis patients.

	T-IS			F-IS			T-PCS			F-PCS		
	Univariate/Multivariate			Univariate/Multivariate			Univariate/Multivariate			Univariate/Multivariate		
	analyses			analyses			analyses			analyses		
	B	(95% CI)	*P*	B	(95% CI)	*P*	B	(95% CI)	*P*	B	(95% CI)	*P*
**Gender/male**	-0.81	-2.04~0.43	0.198	-0.01	-0.03~0.02	0.535	-1.06	-3.73~1.62	0.434	-0.08	-0.18~0.02	0.123
**Age(yrs)**	0.07	0.02~0.12	0.011	0	0~0	0.153	0.2	0.09~0.31	p<0.001	0	0~0.01	0.075
							0.14	0.03~0.26	0.018^M^			
**CKD stages**	1.15	0.61~1.69	p<0.001	0.02	0.01~0.03	p<0.001	2.25	1.06~3.44	p<0.001	0.08	0.04~0.13	0.001
**Liver cirrhosis**	-0.79	-1.62~0.04	0.063	-0.01	-0.03~0	0.089	-2.29	-4.06~-0.52	0.012	-0.05	-0.12~0.02	0.157
							-1.95	-3.81~-0.09	0.040^M^			
**SBP(mmHg)**	0.06	0.02~0.11	0.006	0	0~0	0.001	0.14	0.04~0.23	0.004	0.01	0~0.01	0.001
**DBP(mmHg)**	-0.03	-0.08~0.03	0.381	0	0~0	0.926	-0.08	-0.21~0.04	0.169	0	0~0	0.976
**DM**	0.23	-0.99~1.46	0.707	0.01	-0.01~0.04	0.255	0.88	-1.75~3.52	0.507	0.06	-0.04~0.16	0.232
**HTN**	1.16	-0.11~2.43	0.073	0.03	0.01~0.05	0.009	3.49	0.81~6.18	0.011	0.12	0.02~0.22	0.018
**HBV**	-0.51	-1.79~0.76	0.426	-0.01	-0.03~0.02	0.556	1.03	-1.72~3.77	0.461	-0.01	-0.11~0.1	0.904
**HCV**	0.53	-0.81~1.88	0.433	0.02	0~0.05	0.112	1.96	-0.92~4.85	0.18	0.1	0~0.21	0.061
**Alcohol**	-0.53	-1.77~0.71	0.4	-0.01	-0.03~0.01	0.406	-3.16	-5.76~-0.55	0.018	-0.08	-0.18~0.02	0.105
**Albumin(g/dL)**	0.7	-0.41~1.8	0.212	0.02	0~0.04	0.135	1.95	-0.39~4.3	0.101	0.03	-0.06~0.12	0.515
**Hb(g/L)**	-0.23	-0.52~0.06	0.118	0	-0.01~0	0.222	-0.21	-0.84~0.42	0.504	-0.01	-0.03~0.01	0.349
	-0.1	-0.21~0.01	0.077	0	0~0	0.192	-0.06	-0.3~0.18	0.613	0	-0.01~0	0.346
**BUN(mg/dL)**	0.05	0.02~0.08	p<0.001	0	0~0	p<0.001	0.13	0.07~0.18	p<0.001	0.01	0~0.01	p<0.001
				0.01	0~0.01	p<0.001^M^	0.13	0.07~0.18	p<0.001^M^	0.01	0~0.01	p<0.001^M^
**Cr(mg/dL)**	0.76	0.46~1.06	p<0.001	0.02	0.01~0.02	p<0.001	1.43	0.76~2.1	p<0.001	0.06	0.04~0.09	p<0.001
	0.78	0.31~1.25	0.002^M^									
**eGFR(ml/min)**	-0.03	-0.04~-0.01	p<0.001	0	0~0	p<0.001	-0.06	-0.1~-0.03	p<0.001	0	0~0	p<0.001

^**M**^ **: Multivariate stepwise linear regression analysis**

**Fig 2:**
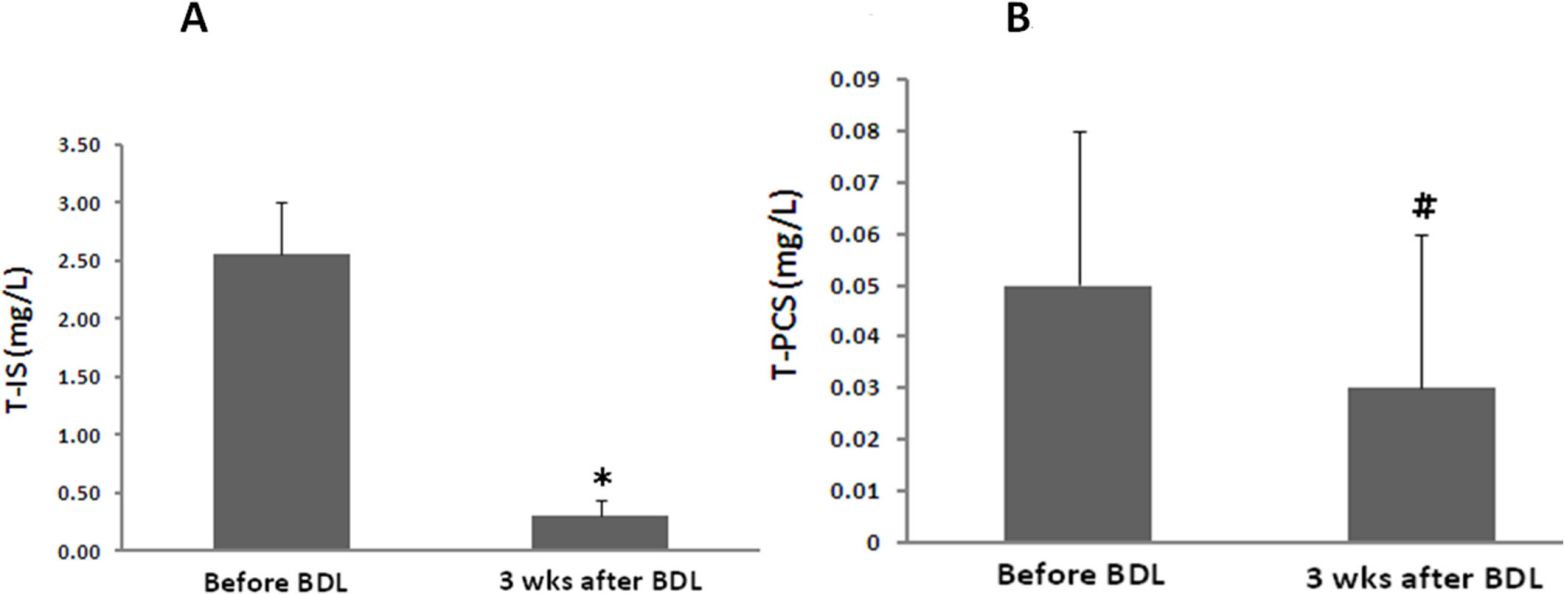
Serum levels of protein-bound uremic toxins (A) T-IS, (B) T-PCS in an animal model with liver failure (*:p<0.01, #:p = 0.17) (N = 5).

**Fig 3:**
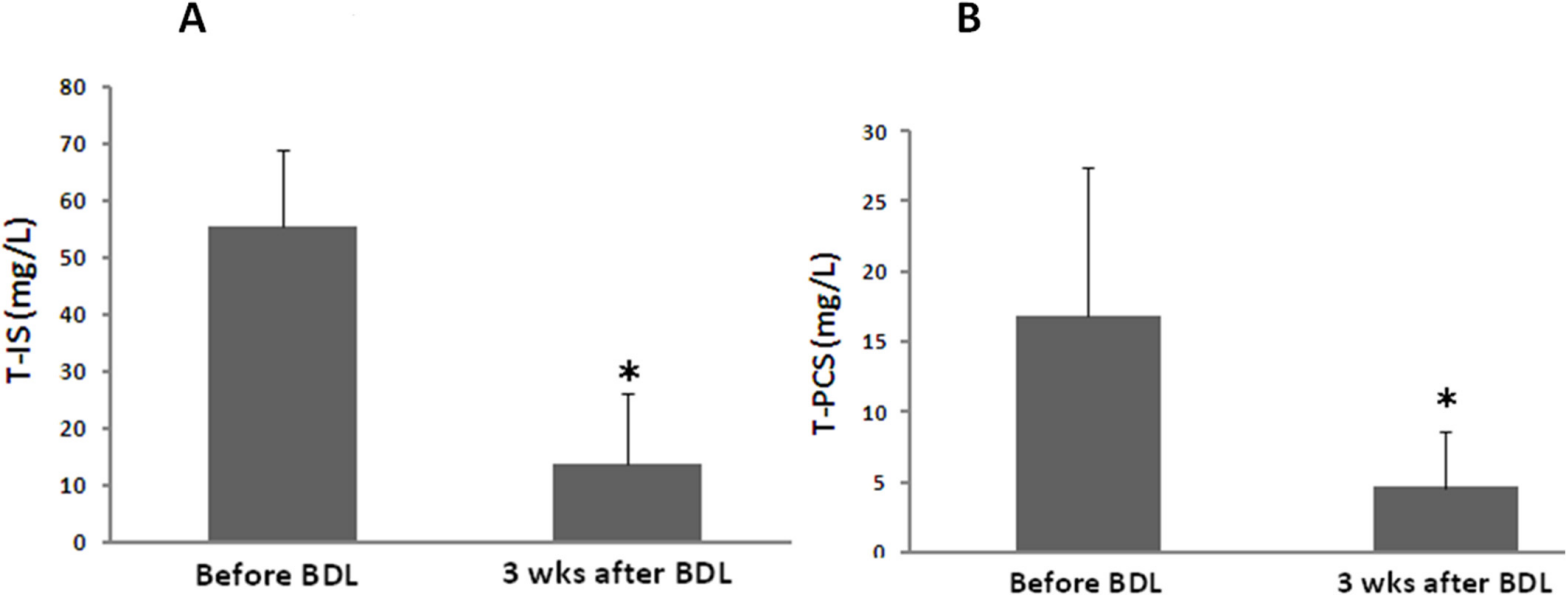
Urine levels of protein-bound uremic toxins (A) T-IS, (B) T-PCS in an animal model with liver failure (*:p<0.05) (N = 5).

In order to confirm the effect of the liver on serum protein-bound uremic toxins, an animal model with CBD ligation was used for the study. The liver function of the study group increased dramatically as compared to the control group (AST: 81.2 ±11.4 vs. 804.1 ± 215.6)(ALT: 51.5 ± 18.6 vs. 154.7 ± 47.6). There was a significant reduction of serum T-IS (2.5±0.5 vs. 0.3±0.1 mg/L, p<0.01) (N = 5) concentrations before and three weeks after bile duct ligation (Fig 2A). The T-PCS level was extremely low before and after surgery, and it did not demonstrate a statistically significant change (0.05 ± 0.03 vs. 0.03 ± 0.04 mg/L, p = NS) (N = 5) (Fig 2B). The urine levels of T-IS and T-PCS were also markedly decreased in rats with liver failure (55.4 ± 13.1 vs. 12.2 ± 13.5, 16.9 ± 10.1 vs. 4.9 ± 3.2 mg/L, respectively) (*p*<0.05) (N = 5) (Fig 3).

## Discussion

Our results showed that kidney function was a predominant factor in determining serum IS and PCS levels in patients with early liver cirrhosis. However, this effect was not present in patients with advanced liver cirrhosis. The two toxin concentrations did not increase in patients with advanced liver and kidney failure. Thus, we demonstrated that in addition to the kidneys, the liver function was an independent factor affecting serum IS and PCS levels.

In recent years, a growing number of publications confirmed the toxicity of IS and PCS from in vitro studies and supported their role in vascular and renal disease progression, contributing to adverse outcomes in CKD patients. [6–8, 12–18]. Thus, this evidence indicated that IS and PCS were not only a vascular toxin but were also nephrotoxins. As we know, the two toxins were generated from the gastrointestinal tract. Indole was the precursor of IS from the fermentation of tryptophan by intestinal flora. [22]. Nevertheless, fermentation of the amino acid tyrosin generates 4-hydroxylphenylacetic acid, which is decarboxylated to p-cresol or demethylated to phenol. [23]. These precursors were absorbed, passing through the intestinal mucosa, before being further metabolised by a hepatic cytosolic sulfotransferase and glucuronyltransferase to indoxyl sulfate, p-cresyl sulfate or p-cresyl glucuronide, respectively. Subsequently, the uremic solutes were secreted to systemic circulation, and were finally removed by renal tubular secretion. [13,14]. Thus, serum IS and PCS in vivo might be determined by the rate of the generation and accumulation of toxic end products from the gut, intestinal flora, liver and kidney.

A previous report by Aronov et al. confirmed the colonic origin of IS and PCS by comparing HD patients with and without a colon. [24]. In addition, indoxyl-β-D-glucuronide, 5-hydroxyindole and α-phenylacetyl-L-glutamine were also identified as colon-derived uremic solutes. The modulation of gut microbiota was regarded as being different approaches to the same theory. One study evaluating the effects of probiotics on serum uremic solutes in a HD cohort [25] showed that serum levels of IS were reduced, probably due to the lower intestinal production of toxins. The use of synbiotics was reported to decrease serum PCS levels in HD patients. [26]. One another small study also suggested that the prebiotic oligofructose-inulin could significantly reduce PCS generation and serum concentrations in HD patients. [27]. The researcher’s findings indicated that changing the intestinal microbiota was an alternative way of affecting IS and PCS concentrations. This evidence supports the thesis of gut-kidney axis in patients with CKD.

Advanced chronic liver disease is responsible for a significant number of physiological changes affecting circulation and kidney perfusion. These changes are associated with primary and secondary kidney disease and have a marked impact on survival. [28]. The glomerular filtration rate was decreased in patients with advanced liver disease, especially those with hepatoranal syndrome. [29]. However, there are very few studies that have investigated whether or not the liver is also an essential organ affecting production of protein-bound uremic solutes. From our results, IS and PCS levels were increased in patients with CKD and early liver cirrhosis. However, the two solutes were lower in CKD with advanced liver cirrhosis. The results indicated that the effect of toxin accumulation from kidney failure was counteracted by liver failure. Studies using animal models with liver failure also confirmed this effect. Thus, we supposed that IS and PCS were significantly dependent on both kidney and liver function.

Several kinds of cytoplasmic sulphotransferases, including phenolsulfotransferases, estrogen sulfotransferase and dehydroepiandrosterone sulphotransferase were expressed in the human liver. Sulfotransferase activity was diminished in liver disease due to a lack of enzyme presence. [30]. Activity of the hepatic enzyme responsible for the modification of these precursors might be lower in the case of liver cirrhosis, leading to a lower generation of the two toxins, though we do not have direct evidence of this point. However, it is unclear on whether having a lower toxin level would have any effect on clinical outcomes. We assume that the gut-kidney axis should be changed to the gut-liver-kidney axis.

There are also two other factors that affect serum toxin concentrations. One is low protein diet, which has been reported to delay CKD progression. [31]. Thus, a low protein diet might affect the serum IS and PCS levels. Patel KP et al. demonstrated that the production of IS and PCS is different in vegetarians versus omnivores. [32]. PCS and IS production rates are markedly lower in vegetarians than they are in individuals that consume an unrestricted diet. The second factor in decreasing toxin concentration is the use of intestinal sorbents, such as AST-120 (Kremezin). Although the effect in delaying kidney function progression was not found to be significant, AST-120 therapy could lower serum IS through the intestinal absorption of indoles and interference of tryptophan metabolism. [33,34].

This study has some limitations. First, the sample size was small and all of the subjects were enrolled from one medical centre. Second, urine samples were not available in our study, so we could not analyse the change of urinary IS and PCS. This analysis could have provided more information about the production of the two solutes. Third, liver function was only assessed and classified by using the Child-Pugh score, rather than using the Indocyanine green (ICG) test. The latter is a more reliable test for determining liver function. In addition, we did not measure the activity of the hepatic cytosolic enzyme for precursor modification. Thus, additional prospective investigations are required to answer these questions.

In summary, our study demonstrated that, in addition to the kidneys, the liver affected serum IS and PCS levels. The gut-kidney axis in CKD should be modified to the gut-liver-kidney axis. Further studies are required to show whether lower levels of uremic toxins have any clinical significance in CKD and liver cirrhosis patients.

## Materials and Methods

### Subjects

This study recruited 115 stable patients with liver cirrhosis and CKD (Stage 1 to Stage 5) receiving treatment from a medical centre. Patients with acute infection, malignancy, with the exception of hepatitis-related hepatoma, or those who were younger than 18 years were excluded. The aetiology of CKD in the patients included type 2 diabetic nephropathy (44.3%) and chronic glomerular nephritis (cGN) (55.7%). The study was performed according to the principles of the Declaration of Helsinki and the ethics committee of the Mackay Memorial Hospital approved the study. All of the patients signed an informed consent form before participating in the study.

### Laboratory assessment

After enrolment in the study, fasting blood samples were obtained for all CKD patients. The estimated GFR (eGFR) was calculated using a modified version of the Modification of Diet in Renal Disease (MDRD) equation, as follows: 175*Scr^-1.154*Age^-0.203*0.742 (if female). The following data were collected: BUN (md/dl), creatinine (mg/dl), haemoglobin (g/dl), haematocrit (%), albumin (g/dl), IS (mg/l), and PCS (mg/l). The bromocresol green method was used for the determination of albumin levels. The Child-Pugh score was used to assess the status of liver cirrhosis, including five clinical measures of liver disease.

Serum IS and PCS were measured with LC-MS/MS (4000 QTRAP, USA). In brief, serum samples were prepared and deproteinised by heat denaturation. The free concentrations of IS and PCS were measured in serum ultrafiltrates, obtained by using Microcon YM-30 separators (Millipore, Billerica, MA, USA). HPLC was performed at room temperature using a dC18 column (3.0 × 50 mm, Atlantis, Waters). The buffers used were (A) 0.1% formic acid and (B) 1mM NH_4_OAc + 0.1% formic acid in 100% acetonitrile. The flow rate was 0.6 mL/min with a 3.5-min gradient cycling from 90% A/10% B to 10% A/90% B. Under these conditions, both PCS and IS were eluted at 2.73 and 2.48 min, respectively. Standard curves for PCS and IS were set at 1, 5, 10, 50, 250, 500 and 1000μg/L; both were processed in the same manner as the serum samples, and they correlated with the serum samples with average r^2^ values of 0.996 ± 0.003. These samples were diluted if the IS or PCS concentrations exceeded the standard curve. Quantitative results were obtained and calculated in terms of their concentrations (mg/L). The sensitivity of this assay was 1μg/L for PCS and 1μg/L for IS.

### Experimental model of liver fibrosis

Male Sprague-Dawley rats (about 260 g) were used in this study. On day two, the common bile ducts were double ligated under halothane anaesthesia. PCS and IS concentrations in the serum and urine were measured before the operation and three weeks after the operation. The local committee for care and use of laboratory animals at the Mackay Memorial Hospital approved this study.

### Statistical analysis

The demographic data were expressed as the mean ± standard deviation (SD). A Pearson’s correlation coefficient was used to analyse the relationship of serum IS or PCS levels with the liver cirrhosis stages and CKD stages. The interaction effect between the CKD stages and liver cirrhosis stages for the increased means of T-IS, F-IS, T-IS and T-PCS were examined using a two-way ANOVA with a Least Squares Means multiple comparison test. In addition, univariate and multivariate (Stepwise) linear regression analyses were used to analyse the relationship between IS or PCS with other independent variables in all of the study patients. In the animal model with liver failure, the serum T-IS and T-PCS levels before and three weeks after surgery were compared using Student t-tests. A value of *P*<0.05 was considered statistically significant. All of the statistical analyses were conducted using the SPSS v.17.0 software programme (SPSS, Chicago, IL).
